# Coenzyme Q and Selenium Co-Supplementation Alleviate Methionine Choline-Deficient Diet-Induced Metabolic Dysfunction-Associated Steatohepatitis in Mice

**DOI:** 10.3390/nu17020229

**Published:** 2025-01-09

**Authors:** Hyewon Choi, Jiwon Choi, Yula Go, Jayong Chung

**Affiliations:** Department of Food & Nutrition, Kyung Hee University, Seoul 02447, Republic of Korea

**Keywords:** coenzyme Q, selenium, metabolic dysfunction-associated steatohepatitis

## Abstract

Background/Objectives: The pathogenesis of metabolic dysfunction-associated steatohepatitis (MASH) is closely associated with increased oxidative stress and lipid peroxidation. Coenzyme Q (CoQ) and selenium (Se) are well-established antioxidants with protective effects against oxidative damage. This study aimed to investigate the effects of CoQ and Se in ameliorating MASH induced by a methionine choline-deficient (MCD) diet in mice. Methods: C57BL/6J male mice were fed either a methionine choline-sufficient (MCS) or MCD diet and treated with vehicle, CoQ (100 mg/kg), Se (158 μg/kg), or their combination (CoQ + Se) for 4 weeks. Results: The MCD diet significantly increased hepatic steatosis, inflammation, and fibrosis compared to MCS controls. Treatment with CoQ and Se, particularly in combination, markedly reduced the MAFLD activity score, hepatic inflammation, and fibrosis. Combined supplementation of CoQ and Se significantly decreased serum alanine aminotransferase and aspartate aminotransferase levels and hepatic TG and cholesterol concentrations. CoQ and Se effectively mitigated hepatic oxidative stress by enhancing catalase and superoxide dismutase activities, increasing glutathione peroxidase (GPX) activity, and restoring the GSH/GSSG ratio. Lipid peroxidation markers, such as malondialdehyde and 4-hydroxynonenal, were significantly reduced. Furthermore, the expression of ferroptosis-related markers, including acyl-CoA synthetase long-chain family member 4, arachidonate 12-lipoxygenase, and hepatic non-heme iron content, was significantly downregulated, while GPX4 expression was upregulated by combined CoQ and Se treatment. Conclusions: CoQ and Se synergistically alleviate MASH progression by reducing oxidative stress and lipid peroxidation, which may contribute to the suppression of ferroptosis. Combined CoQ and Se supplementation demonstrates therapeutic potential for managing MASH and related liver injury.

## 1. Introduction

Metabolic dysfunction-associated steatotic liver disease (MASLD) is among the most prevalent chronic liver conditions, affecting roughly 25% of the global population [1]. Metabolic dysfunction-associated steatohepatitis (MASH) is a more severe form of MASLD characterized by hepatic steatosis, inflammation, and fibrosis. MASH can lead to poor clinical outcomes, including cirrhosis and hepatocellular carcinoma and is an independent risk factor for increased liver-related mortality [2]. Therefore, it is important to prevent the progression of MASH and alleviate the associated liver damage. MASLD and MASH are newly established terms replacing nonalcoholic fatty liver disease (NAFLD) and nonalcoholic steatohepatitis (NASH), respectively [3].

The pathogenesis of MASH is multifactorial, with the widely accepted ‘multiple hit’ theory proposing that the disease is initiated by lipid accumulation in hepatocytes, which induces insulin resistance and makes the liver susceptible to additional damaging factors [4]. Among these factors, oxidative stress and lipid peroxidation are central to MASH progression. Lipid accumulation in hepatocytes elevates oxidative metabolism, leading to excessive production of reactive oxygen species (ROS) that overwhelm the liver’s antioxidant defenses. This oxidative stress causes significant damage to cellular components, including DNA, proteins, and lipids, thereby aggravating hepatocyte dysfunction and injury. Simultaneously, lipid peroxidation, driven by ROS, generates toxic byproducts such as malondialdehyde (MDA) and 4-hydroxynonenal (4-HNE), which exacerbate inflammation and promote hepatocellular death. These mechanisms activate immune responses and fibrogenic pathways, driving the transition from simple steatosis to inflammation and fibrosis [5]. Moreover, recent evidence highlights the role of ferroptosis—a type of cell death characterized by iron-dependent lipid peroxidation—in MASH progression [6,7], underscoring the critical contribution of oxidative stress and lipid peroxidation as key drivers of the disease.

Coenzyme Q (CoQ) and selenium (Se) are essential components of the cellular antioxidant defense system. CoQ, also known as ubiquinone, is a lipid-soluble antioxidant that directly scavenges ROS within cell membranes. It has been shown to protect against liver injuries caused by oxidative stress, such as carbon tetrachloride (CCl_4_)- and acetaminophen-induced hepatotoxicity in animal studies [8,9]. However, studies investigating the relationship between CoQ and MASH are scarce. Similarly, Se is an essential component of selenoproteins, many of which exhibit antioxidant properties. One notable example is glutathione peroxidase (GPX), whose activity is dependent on Se. Se supplementation has shown beneficial effects on liver function in models of oxidative stress-induced damage, such as CCl_4_-treated rats [10]. However, high doses of Se have been associated with potential adverse effects on liver function [11,12], making its protective role in liver diseases inconclusive. Additionally, both CoQ and Se have been implicated in the regulation of ferroptosis, a form of cell death characterized by iron-dependent lipid peroxidation. CoQ serves as a substrate for ferroptosis suppressor protein 1 (FSP1), and Se acts as a co-factor for GPX4, both of which are key regulators of ferroptosis [13,14]. Despite these antioxidant roles and their involvement in ferroptosis regulation, the effects of CoQ, Se, or their combination on the progression of MASH remain unexplored. Thus, the objective of this study was to evaluate the protective effects of CoQ, Se, and their combination on the progression of MASH and to elucidate the mechanisms underlying these effects.

## 2. Materials and Methods

### 2.1. Animals and Experimental Design

C57BL/6J male mice (4 weeks old) were purchased from Central Lab Animal, Inc. (Seoul, Republic of Korea) and maintained at 22 ± 2 °C temperature, 50 ± 10% humidity on a 12 h dark/light cycle. After an adaptation period, the animals were randomly assigned to one of eight groups (*n* = 10/group). Four groups were given a methionine choline-deficient (MCD) diet, and the other four groups were fed a methionine choline-sufficient (MCS) diet. At the same time, each diet group was treated with corn oil (vehicle), CoQ (100 mg/kg), Se (158 μg/kg), or both (CoQ + Se) by oral gavage once a day for 4 weeks. The doses used in this study were selected based on findings from previous studies [6,15]. All diets were purchased from Research Diets, Inc. (New Brunswick, NJ, USA). At the end of the feeding period, the mice were killed under carbon dioxide (CO_2_) anesthesia. Liver tissue samples were collected and either fixed in 10% neutral buffered formalin or snap-frozen in liquid nitrogen and stored at −80 °C until further analyses. All protocols and procedures were approved by the Kyung Hee University Institutional Animal Care and Use Committee (#KHSASP-21-341).

### 2.2. Serum and Hepatic Biochemical Parameters

Serum aspartate aminotransferase (AST) and alanine aminotransferase (ALT) activities were measured using assay kits (#AM102-K and #AM103-K, respectively, Asan Pharmaceutical Co., Hwa-sung, Kyung-gi, Republic of Korea). Hepatic triglyceride (TG) and total cholesterol (TC) concentrations were measured using assay kits (#AM157S and #AM202, Asan Pharmaceutical Co.). Hepatic MDA contents were measured using an assay kit (#MAK085, Sigma-Aldrich^®^, Saint Louis, MO, USA). Hepatic non-heme iron contents were measured according to the method described by Choi et al. [16]. Hepatic glutathione (GSH) and oxidized glutathione (GSSG) contents, hepatic catalase (CAT), superoxide dismutase (SOD), and GPX activity were measured using commercially available kits (Cayman Chemical, Ann Arbor, MI, USA). All procedures were performed according to the manufacturer’s protocols.

### 2.3. Histological Analysis

Fixed liver tissues, embedded in paraffin, were sliced into 5 μm thick slices and stained with hematoxylin and eosin (H&E) (KP&T Co., Ltd., Cheong-ju, Chung-buk, Republic of Korea). The MASLD activity score (MAS) score was evaluated by examining the degree of steatosis, lobular inflammation, and hepatocellular ballooning [16]. To assess liver fibrosis, Sirius Red staining was performed. Then, the area of the red part as a percentage of the total area was quantified using ImageJ software version 1.53 (NIH, Bethesda, MD, USA). The average of the five fields was calculated. All stained-liver sections were observed under an optical microscope (Olympus, Tokyo, Japan) at 200× magnification.

### 2.4. Quantitative Real Time RT-PCR

Total RNA was extracted from 25 mg of liver tissue using the RNAiso Plus reagent (Takara Bio Inc., Shiga, Japan). For cDNA synthesis, 100 ng of total RNA was reverse transcribed using the PrimeScript™ RT reagent Kit (Takara Bio Inc.). Real-time reverse transcription polymerase chain reaction (RT-PCR) was performed to amplify specific genes with the SYBR Premix Ex Taq kit (Takara Bio Inc.). mRNA expression levels were quantified relative to the control group using the 2^−ΔΔCt^ method.

### 2.5. Western Blot Analysis

Liver tissue (50 mg) was lysed in 200 μL of lysis buffer with protease inhibitors and centrifuged at 17,000 rpm for 15 min at 4 °C. Protein concentrations were measured using a Protein Quantification Kit (Thermo Fisher Scientific Inc., Waltham, MA, USA). Lysates were separated by Sodium Dodecyl Sulfate Polyacrylamide Gel Electrophoresis (SDS-PAGE), transferred to a Polyvinylidene Fluoride (PVDF) membrane, blocked, and incubated overnight at 4 °C with primary antibodies (anti-GPX4 (Abcam, Cambridge, UK), anti-4HNE (R&D systems, Minneapolis, MN, USA), anti-ferritin, anti-acyl-CoA synthetase long-chain family member 4 (ACSL4), anti-12-lipoxygenase (LOX), and anti- glyceraldehyde-3-phosphate dehydrogenase (GAPDH) (all from Santa Cruz Biotechnology, Dallas, TX, USA)). Membranes were incubated with a secondary antibody for 1 h and developed with Enhanced Chemiluminescence solution (Bio-Rad, Hercules, CA, USA). Bands were visualized using a ChemiScope (Clinx Science Instruments Co., Shanghai, China) and quantified.

### 2.6. Statistical Analysis

All data were analyzed using SAS 9.4 software and expressed as mean ± SEM. One-way Analysis of Variance (ANOVA) with post-hoc Duncan’s multiple range test was performed to test the significant difference among groups. *p*-values less than 0.05 were considered statistically significant.

## 3. Results

### 3.1. CoQ and Se Alleviate Liver Injury in MASH Mice Induced by MCD Diet

**Figure 1:**
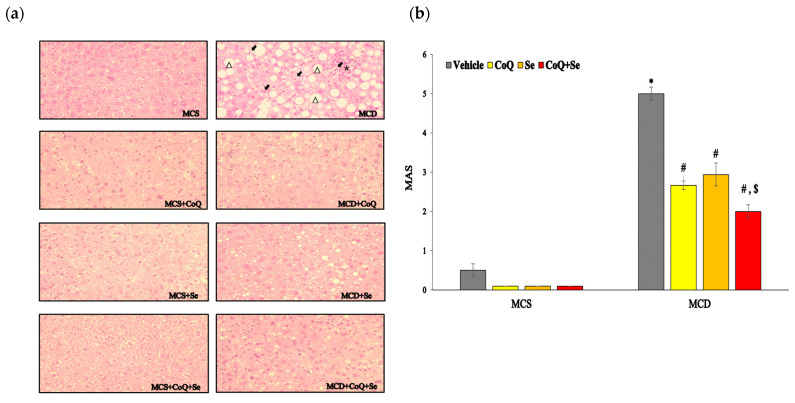

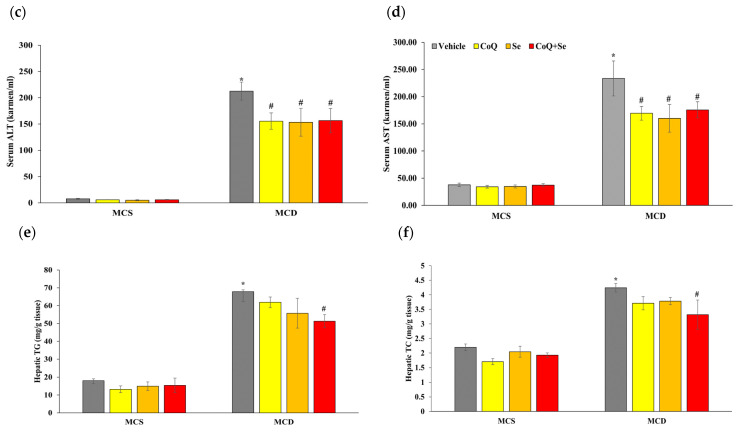
Effects of CoQ and Se treatment on liver injury in MCD diet-induced MASH mice. (**a**) Liver sections stained with H&E (steatosis (Δ), lobular inflammation (*), and ballooning degeneration (→)), (**b**) MAS score, (**c**) Serum ALT activity, (**d**) Serum AST activity, (**e**) Hepatic TG concentration, (**f**) Hepatic total cholesterol (TC) concentration. Data are shown as mean ± SEM. * *p* < 0.05 vs. MCS vehicle, ^#^ *p* < 0.05 vs. MCD vehicle, ^$^ *p* < 0.05 vs. MCD + CoQ.

In the histopathological analysis of H&E-stained liver sections, mice on the MCD diet exhibited a significant increase in both the size and number of fat droplets, along with pronounced inflammatory cell infiltration and ballooning degeneration, compared to those on the MCS diet control (Figure 1a). Conversely, treatments with CoQ, Se, or their combination markedly reduced hepatic steatosis, decreased inflammatory foci, and improved ballooning degeneration. The MAS scores were significantly lower in the CoQ, Se, and CoQ + Se groups compared to the MCD group, with the CoQ + Se group reaching the lowest scores among the four MCD diet-fed groups (Figure 1b). Serum levels of ALT and AST, which were significantly elevated in the MCD group relative to the MCS group, were notably reduced by treatments with CoQ, Se, or CoQ + Se (Figure 1c,d). Hepatic TG and TC concentrations were considerably higher in the MCD group than in the MCS group (Figure 1e,f). While there was a trend towards reduced hepatic TG and total cholesterol levels with CoQ or Se treatment alone, these changes were not statistically significant. However, the combination of CoQ and Se significantly lowered both hepatic TG and cholesterol concentrations compared to the MCD group.

### 3.2. CoQ and Se Alleviate Inflammation and Fibrosis in MASH Mice Induced by MCD Diet

**Figure 2:**
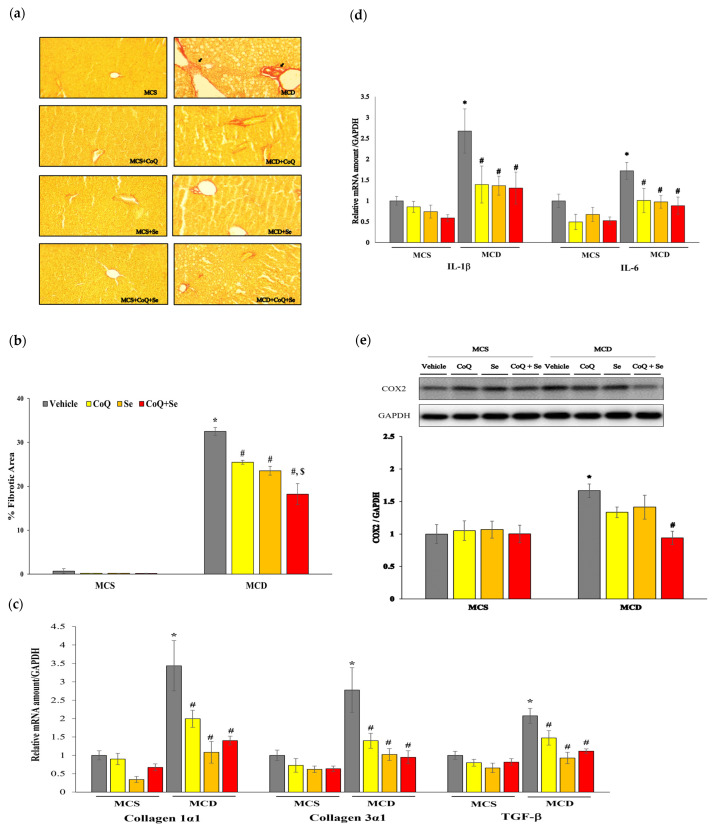
Effects of CoQ and Se treatment on hepatic inflammation and fibrosis in MCD diet-induced MASH mice. (**a**): Liver sections stained with Sirius Red staining (200× magnification); The arrow indicates collagen staining, (**b**) % Fibrotic area, (**c**) the mRNA levels of fibrotic markers, (**d**) Hepatic mRNA levels of pro-inflammatory cytokines, (**e**) Hepatic protein levels of COX2 with representative blot (upper panel). Data are shown as mean ± SEM. * *p* < 0.05 vs. MCS vehicle, ^#^ *p* < 0.05 vs. MCD vehicle, ^$^ *p* < 0.05 vs. MCD + CoQ.

The degree of fibrosis was evaluated using Sirius Red staining of liver sections. Collagen staining was much more pronounced in the MCD group compared to the MCS control group but was significantly reduced in the groups treated with CoQ, Se, or CoQ + Se (Figure 2a,b). The combination treatment further decreased collagen staining compared to treatment with CoQ or Se alone. The mRNA expression levels of Collagen 1α1, Collagen 3α1, and transforming growth factor-β (TGF-β), indicators of fibrosis, were significantly higher in the MCD group compared to the MCS group, and were substantially reduced following CoQ, Se, and CoQ + Se treatments (Figure 2c).

In addition, the mRNA levels of pro-inflammatory cytokine related genes such as interleukin-1β (IL-1β) and interleukin-6 (IL-6) were significantly increased in the MCD group compared to the MCS group. In contrast, treatment with CoQ, Se, or CoQ + Se markedly downregulated both IL-1β and IL-6 mRNA levels. Moreover, the level of cyclooxygenase-2 (COX2) protein expression, which mediates inflammatory responses, was significantly increased in the MCD vehicle group. The combination of CoQ and Se treatment, but not CoQ or Se alone, significantly down-regulated COX2 protein expression compared to the MCD group.

### 3.3. CoQ and Se Alleviate Hepatic Oxidative Stress in MCD Diet-Induced MASH Mice

Antioxidant enzymes such CAT, SOD, and GPX play crucial roles in defending against ROS. In this study, the MCD diet significantly reduced CAT and SOD activities compared to the MCS control group, indicating induced oxidative stress (Table 1). Although treatment with CoQ or Se alone showed a trend of increasing CAT and SOD activities, these changes were not statistically significant. However, the combination of CoQ and Se significantly enhanced both CAT and SOD activities, demonstrating a synergistic effect that effectively counteracts the oxidative stress from the MCD diet (Table 1). GPX activity was higher in the MCD group compared to the MCS control, as a response to increased oxidative stress. Administration of CoQ or Se alone further enhanced GPX activity, with their combination resulting in a statistically significant increase compared to the MCD group, highlighting their potent combined effect in enhancing the GSH antioxidant defense system.

Furthermore, the MCD diet significantly decreased the GSH/GSSG ratio, a key marker of cellular redox balance, but combined supplementation of CoQ and Se substantially improved this ratio to levels comparable to the MCS group, emphasizing their capacity to restore a favorable redox balance and mitigate oxidative damage.

### 3.4. CoQ and Se Inhibit Hepatic Lipid Peroxidation in MCD Diet-Induced MASH Mice

**Figure 3:**
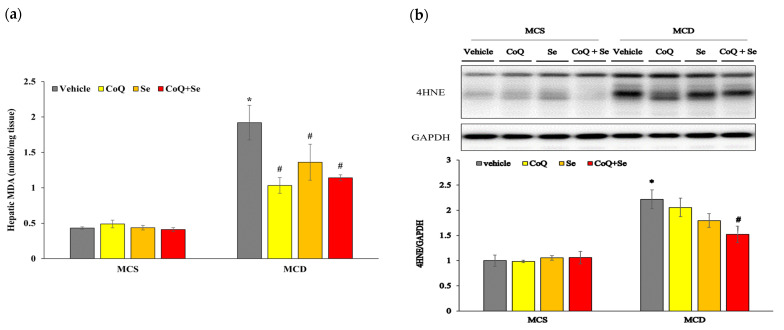
Effects of CoQ and Se treatment on hepatic lipid peroxidation in MCD diet-induced MASH mice. (**a**) Hepatic MDA concentration, (**b**) Hepatic protein levels of 4-HNE with representative blot (upper panel). Data are shown as mean ± SEM. * *p* < 0.05 vs. MCS vehicle, ^#^ *p* < 0.05 vs. MCD vehicle.

The increase in hepatic lipid peroxidation is closely linked to the onset and progression of MASH. We investigated whether CoQ, Se, or their combination could mitigate these effects in MCD diet-induced MASH mice. The data showed that the MCD diet significantly increased hepatic MDA levels compared to the MCS control diet (Figure 3a). Levels of 4-HNE were also significantly elevated in the MCD group compared to the MCS group (Figure 3b). In contrast, treatment with CoQ, Se, or both significantly decreased hepatic MDA levels induced by the MCD diet. Additionally, the combination of CoQ and Se significantly reduced hepatic 4-HNE levels. As MDA and 4-HNE are key biomarkers of lipid peroxidation, these findings indicate that CoQ and Se treatments significantly ameliorated hepatic lipid peroxidation in MASH mice.

### 3.5. CoQ and Se Inhibit Changes in Ferroptosis Related Markers in the Liver of MCD Diet-Induced MASH Mice

**Figure 4:**
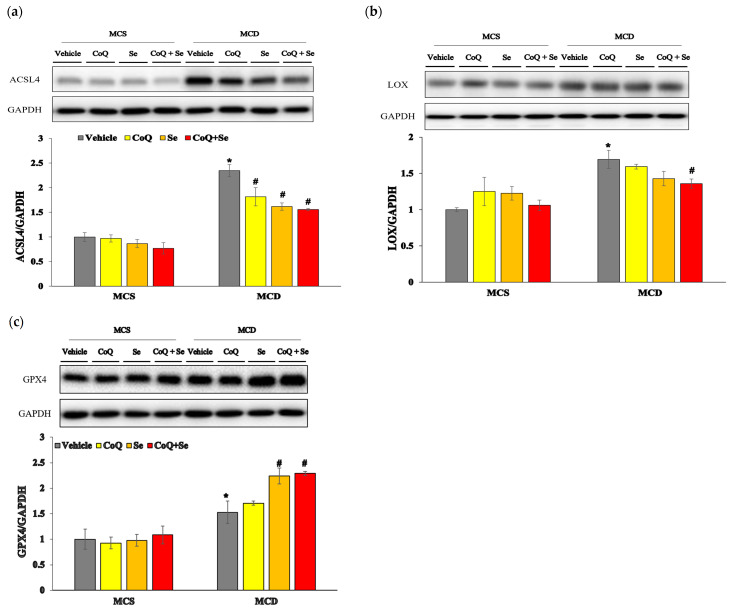
Effects of CoQ and Se treatment on hepatic ferroptosis markers in MCD diet-induced MASH mice. Hepatic protein levels of (**a**) ACSL4, (**b**) LOX, and (**c**) GPX4. Data are shown as mean ± SEM. * *p* < 0.05 vs. MCS vehicle, ^#^ *p* < 0.05 vs. MCD vehicle.

Emerging evidence suggests that ferroptosis, a type of cell death characterized by iron-dependent lipid peroxidation, plays a critical role in the pathogenesis of MASH. To further investigate the molecular mechanisms underlying the effects of CoQ and Se in reducing lipid peroxidation and mitigating liver injury, we examined markers of ferroptosis in this study. The expression level of the ACSL4 protein was significantly increased in the MCD group compared to the MCS control group; however, treatment with CoQ, Se, or their combination (CoQ + Se) resulted in a significant decrease in ACSL4 protein expression (Figure 4a). Similarly, the expression of the LOX protein was markedly elevated in the MCD group compared to the MCS vehicle group. Notably, only the combination treatment of CoQ and Se led to a significant reduction in LOX protein expression (Figure 4b). Furthermore, the protein levels of GPX4 were increased in the MCD group compared to the MCS vehicle group. GPX4 expression was further elevated in the Se alone and CoQ + Se treatment groups compared to the MCD group (Figure 4c).

**Figure 5:**
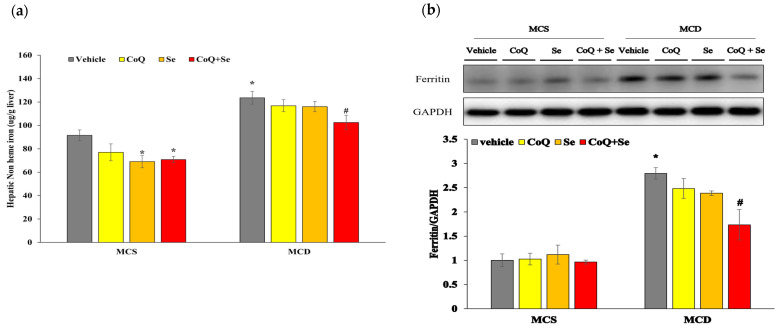
Effects of CoQ and Se treatment on hepatic non-heme iron concentrations and hepatic ferritin protein level in MCD diet-induced MASH mice. (**a**) Hepatic non-heme iron concentration, (**b**) Hepatic protein levels of ferritin. Data are shown as mean ± SEM. * *p* < 0.05 vs. MCS vehicle, ^#^ *p* < 0.05 vs. MCD vehicle.

We also evaluated changes in hepatic iron content by measuring non-heme iron concentrations and ferritin levels, which is a cellular iron storage protein. The non-heme iron concentration was significantly higher in the MCD group compared to the MCS vehicle group, whereas treatment with CoQ + Se significantly reduced non-heme iron levels compared to the MCD group (Figure 5a). Among the groups fed the MCS diet, Se alone and CoQ + Se treatments resulted in significantly lower non-heme iron concentrations compared to the MCS vehicle group. Ferritin protein levels were significantly increased in the MCD diet groups compared to the MCS control group. In contrast, ferritin protein levels showed a significant decrease in the group treated with the combination of CoQ and Se compared to the MCD group (Figure 5b).

## 4. Discussion

MASH, a severe form of MASLD, is a major global health concern due to its association with hepatic inflammation, fibrosis, and progression to cirrhosis or hepatocellular carcinoma. Despite its growing prevalence, effective therapeutic options remain limited. In this study, we provide compelling evidence that co-supplementation with CoQ and Se effectively ameliorates the progression of MASH induced by an MCD diet in mice. The combined treatment significantly alleviated hepatic steatosis, inflammation, and fibrosis. These effects were associated with the suppression of oxidative stress, lipid peroxidation, and markers of ferroptosis.

The pathogenesis of MASH is closely associated with increased oxidative stress [17]. Depletion of GSH and CoQ and decreases in the activity of certain antioxidant enzymes such as CAT and SOD were reported in patients with MASH, which were correlated with the severity of the disease [18]. One major consequence of oxidative stress is lipid peroxidation, where ROS attack polyunsaturated fatty acids in cell membrane phospholipids. This process produces toxic byproducts like MDA and 4-HNE, which can activate Kupffer and hepatic stellate cells, leading to inflammation and the onset of fibrogenesis. In this study, we observed a significant reduction in CAT and SOD activities, along with a decreased GSH/GSSG ratio, in the liver tissues of MCD-diet-fed mice compared to MCS controls. Hepatic levels of MDA and 4-HNE were also markedly elevated in the MCD group, indicating increased oxidative stress and lipid peroxidation due to MCD-diet feeding. Conversely, CoQ and Se co-supplementation significantly enhanced CAT and SOD activities, restored the GSH/GSSG ratio to levels comparable to those of the MCS controls, and significantly reduced MDA and 4-HNE levels, contributing to improvements in NAS, inflammation, and fibrosis. CoQ prevents both the initiation and propagation of lipid peroxidation [19]. Additionally, Se-dependent GPX4 is the major antioxidant enzyme known to catalyze the conversion of toxic lipid hydroperoxide into non-toxic lipid alcohols [20]. While CoQ and Se have each been individually reported to exhibit hepatoprotective effects [8,9,10], their roles in MASH have not been well established. This study provides novel evidence of the synergistic effects of CoQ and Se co-supplementation in mitigating MASH progression.

Recent studies have emphasized the critical role of ferroptosis in the progression of MASH [11,12,13]. In line with this, our study demonstrated significant increases in hepatic expression of ferroptosis markers, including ACSL4 and LOX, in the MCD group compared to MCS controls. These changes were accompanied by elevated hepatic iron content and markedly increased levels of MDA and 4-HNE, key indicators of lipid peroxidation. Similar findings were reported by [7], who showed that iron and lipid ROS accumulation in the liver of MCD-diet-fed mice was alleviated by ferroptosis inhibitors, leading to reductions in inflammation, fibrosis, and liver injury. These observations suggest that ferroptosis contributes to MCD-diet-induced MASH progression, as corroborated by our findings.

To further elucidate the molecular mechanisms underlying the hepatoprotective effects of CoQ and Se against MASH progression, we investigated changes in ferroptotic markers. Notably, the combination of CoQ and Se significantly reduced hepatic LOX protein levels, accompanied by marked decreases in lipid peroxidation, as indicated by lower MDA and 4-HNE levels. In contrast, CoQ treatment alone did not significantly alter hepatic LOX protein or 4-HNE levels. Interestingly, treatment with Se or CoQ + Se induced a more than two-fold increase in hepatic GPX4 expression compared to MCS controls. GPX4, a pivotal enzyme for eliminating lipid peroxides, is essential for ferroptosis regulation; its deletion leads to lipid peroxide accumulation and ferroptosis, whereas its overexpression suppresses ferroptosis [20,21]. Se has been shown to transcriptionally upregulate selenoproteins, including GPX4, protecting cells from ferroptotic stimuli [13]. Additionally, Se treatment has been shown to increase GPX4 protein levels in palmitic acid-treated hepatocytes, preventing ferroptotic cell death and promoting cell survival [6]. In our study, the induction of GPX4 was associated with the suppression of ACSL4 and LOX expression, suggesting that CoQ and Se treatment mitigates MASH progression, at least in part, by suppressing ferroptosis.

Interestingly, Se treatment alone induced GPX4 expression to a level comparable to those achieved by CoQ + Se treatment; however, hepatic 4-HNE level was further decreased in the CoQ + Se treatment group compared to the Se-alone group. This suggests that CoQ may provide additional antioxidant activity beyond GPX4 induction mediated by Se. Alternatively, CoQ might suppress ferroptosis via a GPX4-independent pathway. The CoQ oxidoreductase FSP1, for example, has been shown to confer resistance to ferroptosis and acts in parallel to GPX4 in inhibiting ferroptosis [14,22]. Therefore, CoQ and Se may synergistically suppress ferroptosis through activation of FSP1- and GPX4-dependent pathways, respectively. Future studies are necessary to elucidate the dose-response relationships and further identify the optimal combinations of CoQ and Se supplementation to maximize therapeutic efficacy.

In this study, hepatic non-heme iron concentrations and hepatic ferritin levels were significantly increased in the MCD group compared to the MCS controls. Consistent with this finding, Palladini et al. [23] also reported an increase in iron levels in both serum and tissues during the progression from steatosis to steatohepatitis in an MCD-diet rat model. Excessive iron can induce oxidative stress through the Fenton reaction and has been shown to aggravate liver injury associated with MASLD [24,25]. In addition, iron has been suggested to enhance lipid peroxidation through the activation of enzymes such as LOX [26]. Moreover, the administration of deferoxamine (an iron-chelating agent) has been found to reduce MASH severity in MCD-fed mice [6], indicating that inhibiting iron accumulation may suppress disease progression and associated liver injury. In the current study, the co-treatment of CoQ and Se significantly decreased hepatic ferritin, an iron storage protein, and markers of hepatic iron content, suggesting that this combination is effective in reducing hepatic iron levels. Since synthetic compounds like deferoxamine have limitations such as low oral bioavailability and a short plasma half-life, our findings suggest that CoQ and Se could be alternatives to prevent iron-related diseases.

The mechanisms by which CoQ and Se treatment reduce hepatic iron levels are unclear. A recent study demonstrated that nobiletin, a flavonoid with antioxidant properties, alleviated myocardial ischemia-reperfusion injury by reducing the expression of nuclear receptor coactivator 4 (NCOA4) [27]. NCOA4 is integral to ferritinophagy, an autophagic pathway responsible for ferritin degradation and iron release. Interestingly, erastin, which generates lipid ROS, has been shown to decrease NCOA4 levels, disrupting ferritinophagy and resulting in iron accumulation and increased ferritin levels [28]. Whether CoQ or Se influences hepatic iron levels through modulation of NCOA4 expression or activity warrants further investigation in future studies.

In this study, we demonstrated that CoQ + Se treatment significantly reduced hepatic triglyceride and total cholesterol concentrations. Hepatic lipid accumulation is known to trigger oxidative stress, and the severity of hepatic steatosis is closely associated with the progression of liver injury and fibrosis. Supporting this, previous studies have shown that CoQ improves MASLD in mice by enhancing fatty acid oxidation and inhibiting fatty acid synthesis via activation of the AMPK pathway [29,30]. However, clinical studies have reported inconsistent results, with CoQ10 intervention showing no significant effects on lipid profiles [31]. Similarly, Se has been reported to regulate lipid metabolism through AMPK activation, but its effects on lipogenesis remain conflicting, with studies showing both increases and decreases in lipid synthesis [12,32]. As demonstrated in this study, the combined use of CoQ and Se may provide a more effective strategy for regulating lipid metabolism and reducing hepatic lipid accumulation.

## 5. Conclusions

In conclusion, this study demonstrates that co-supplementation with CoQ and Se effectively alleviates MCD diet-induced MASH. The combined treatment significantly reduced hepatic steatosis, inflammation, and fibrosis. Mechanistically, CoQ and Se enhanced antioxidant defenses by increasing CAT and SOD activities, restoring the GSH/GSSG ratio, and reducing lipid peroxidation, as evidenced by lower levels of MDA and 4-HNE in liver tissues. These changes were associated with the downregulation of pro-inflammatory cytokines such as IL-1β and IL-6, as well as fibrosis-related genes, including collagen 1α1, collagen 3α1, and TGF-β. Furthermore, CoQ and Se suppressed proteins involved in ferroptosis, such as ACSL4 and LOX, while upregulating GPX4 expression. These findings suggest the synergistic potential of CoQ and Se co-supplementation as a promising therapeutic strategy for managing MASH and associated liver injury.

## Figures and Tables

**Table 1:** Effects of coenzyme Q (CoQ) and selenium (Se) on hepatic oxidative stress in MASH mice.

	MCS	MCD			
	Vehicle	Vehicle	CoQ	Se	CoQ + Se
CAT activity (U/mg protein)	1045.9 ± 197.5 ^a^	355.1 ± 24.7 ^c^	537.0 ± 54.6 ^bc^	373.5 ± 23.8 ^c^	649.2 ± 78.8 ^b^
SOD activity (U/mg protein)	33.3 ± 1.1 ^a^	20.4 ± 1.3 ^c^	23.0 ± 2.3 ^bc^	24.2 ± 0.8 ^bc^	27.2 ± 0.8 ^ab^
GPX activity (U/mg tissue)	0.1 ± 0.1 ^d^	1.6 ± 0.3 ^bc^	2.1 ± 0.4 ^b^	2.1 ± 0.1 ^b^	4.0 ± 0.5 ^a^
GSH/GSSG	20.0 ± 5.4 ^a^	4.9 ± 0.7 ^c^	7.3 ± 1.5 ^bc^	6.2 ± 2.2 ^c^	16.4 ± 3.9 ^ab^

Data are expressed as the mean ± SEM. Different superscripts represent statistical significance (*p* < 0.05). One-way ANOVA followed by Duncan’s post-hoc test. CAT, catalase; SOD, superoxide dismutase; GPX, glutathione peroxidase; GSH, glutathione; GSSG, oxidized glutathione.

## Data Availability

The original contributions presented in this study are included in the article. Further inquiries can be directed to the corresponding author.

## Abbreviations

The following abbreviations are used in this manuscript:

4-HNE	4-Hydroxynonenal
ACSL4	Acyl-CoA synthetase long-chain family member 4
CAT	Catalase
CoQ	Coenzyme Q
COX2	Cyclooxygenase-2
FSP1	Ferroptosis suppressor protein 1
GPX	Glutathione peroxidase
GSH	Glutathione
GSSG	Oxidized glutathione
LOX	Arachidonate 12-lipoxygenase
MASH	Metabolic dysfunction-associated steatohepatitis
MASLD	Metabolic dysfunction-associated steatotic liver disease
MCD	Methionine choline-deficient
MCS	Methionine choline-sufficient
MDA	Malondialdehyde
NAFLD	Nonalcoholic fatty liver disease
NASH	Nonalcoholic steatohepatitis
NCOA4	Nuclear receptor coactivator 4
Se	Selenium
SOD	Superoxide dismutase
